# Zoological nomenclature in the digital era

**DOI:** 10.1186/1742-9994-10-4

**Published:** 2013-02-04

**Authors:** Alessandro Minelli

**Affiliations:** 1 Department of Biology, University of Padova, Via Ugo Bassi, Padova 58B I 35131, Italy

**Keywords:** Digital publication, International code of zoological nomenclature, International commission on zoological nomenclature, Scientific names of animals, ZooBank

## Abstract

Creation and use of the scientific names of animals are ruled by the International Code of Zoological Nomenclature. Until recently, publication of new names in a work produced with ink on paper was required for their availability. A long awaited amendment to the Code issued in September 2012 by the International Commission on Zoological Nomenclature now allows publication of new names in online-only works, provided that the latter are registered with ZooBank, the Official Register of Animal Names. With this amendment, the rules of zoological nomenclature have been aligned with the opportunities (and needs) of our digital era. However, possible causes for nomenclatural instability remain. These could be completely removed if the Code-compliant publication of new names will be identified with their online registration, under suitable technological and formal (legal) conditions. Future developments of the ZooBank may provide the tool required to make this definitive leap ahead in zoological nomenclature.

## Introduction

Publications containing the description of new species, the proposal of names for new supraspecific taxa, or other acts affecting the application of a given name to a given taxon are different, in an important sense, from ordinary scientific books or papers [1]. This is because introducing a new species name, or otherwise intervening on the use of the scientific names of animals, is not simply a way to translate into words the author’s view on a particular problem of animal taxonomy. The choice of the names by which we refer to the individual species, genera or families in the animal kingdom is ruled by a set of principles known as the *International Code of Zoological Nomenclature*[2] (hereafter, ‘the *Code’*). Thus, in a sense, irrespective of its scientific quality or importance, a publication relevant to zoological nomenclature must be treated as a legal document. As such, it remains on record, virtually, for ever.

Until recently, publication of new names in a work produced with ink on paper was required for their availability. A long awaited amendment to the *Code* issued in September 2012 by the International Commission on Zoological Nomenclature now allows publication of new names in online-only works, provided that the latter are registered with ZooBank, the Official Register of Animal Names. With this amendment, the rules of zoological nomenclature have been aligned with the opportunities (and needs) of our digital era. However, possible causes for nomenclatural instability remain.

I mean, here, instability for intrinsic, purely nomenclatural reasons. These must be kept clearly separate from name changes due to alternative taxonomic views. Nomenclature should provide a unique name for each taxon in the classification, but, as stated in the Preamble to the *Code*, no rule is intended to restrict the freedom of taxonomic thought or actions [2]. How true this eventually is in practice, is an interesting question [3] that has been raised [4] even before an alternative set of rules, the PhyloCode [5], was proposed to provide names for taxonomic entities recognized within a taxonomy that does not accept the ranks, such as the family or the genus, of the traditional Linnaean taxonomy. But this sensible aspect to the relationships between taxonomy and nomenclature will not be discussed here. In this Commentary I will briefly review the requirements for publication of new names and other nomenclatural acts as traditionally fixed in the Code and the main changes introduced with the recent amendment. My focus will be on possible problems remaining after the enforcement of the new rules for publication, to end with suggestions for an operational solution involving a major change in the concept of registration.

### The International Code of Zoological Nomenclature

The need to fix the rules of zoological nomenclature was first addressed by a committee appointed by the British Association for the Advancement of Science. This committee of twelve members, among which Charles Darwin and Richard Owen, produced a document [6] that eventually inspired parallel efforts in other countries [7-9] but essentially remained a reference point for the British scientific community only. Only in the last years of the XIX century the problem was finally tackled at international level, when the third International Congress of Zoology (Leyden 1895) appointed a Commission on Zoological Nomenclature, under the presidency of the French zoologist Raphaël Blanchard. It took several years to get a document eventually approved by the fifth International Congress of Zoology (Berlin 1901) and published thereafter in three official equivalent versions, in French, English and German respectively [10]. Responsibility for the application of the *Règles* and for possible changes to the same remained with the International Commission on Zoological Nomenclature, turned into a permanent body taking authority from the International Congress of Zoology.

In the following decades, a number of amendments to the *Règles* were issued. The most important events in the subsequent history of zoological nomenclature [8,9], however, were the replacement of the old *Règles* by a new *Code*[11] and the eventual extinction, with the nineteenth edition held in Monte Carlo (1972), of the series of international congresses (tentatively resurrected with the Athens edition held in 2000). As a consequence of the death of the parent institution, the International Commission on Zoological Nomenclature (hereafter, ’the Commission’) was eventually put under the umbrella of the International Union of Biological Sciences (IUBS). In the last half of a century, two extensively revised editions the *Code* have been issued [1,12], but even the latter clearly was (and still is) in need of revision.

From this strongly abridged history of zoological nomenclature it is easy to guess that the current *Code* is far from being a set of a few, straightforward rules. This quite likely looks awkward to all those scientists who are users rather than producers of the scientific names of organisms. A couple of paragraphs are thus in order, to present the main issues requiring fixed, internationally agreed rules, and also to explain why these rules cannot be fixed once and forever. The *Code*’s articles need be updated from time to time, not only in respect to the progress in the appreciation of the evolutionary, genetic and ecological structure of biodiversity, but also in respect to the changing practices in issuing and distributing scientific publications.

### The rules and the Commission

The basic aim of the *Code*’s rules is to achieve universality and uniqueness in the choice of names to be applied to the individual species, genera and families recognized by taxonomists. Problems arise whenever different names are found to have been proposed for the same taxon (*synonymy*), or when the same name has been applied to different taxa (*homonymy*).

The main principle to be adopted to solve problems of synonymy or homonymy is the Principle of Priority. Thus, of two synonyms, the older is the valid one; of two homonyms, the older is the valid name for the taxon to which it has been applied, whereas the junior homonym must be replaced by the next available name thus far applied to the relevant taxon, or by a new name, if no other name is available.

In the vast majority of cases, routinary application of the Principle of Priority is adequate to keep zoological nomenclature close to its target of universality and uniqueness, but in the daily practice of taxonomy difficult problems of nomenclature continue to crop up. Examples can be found at the Commission’s website [13]. Many of these difficult cases concern the possible disruptive consequences of a strict application of the Principle of Priority. Imagine we discover today, in a dusty issue of the obscure journal of a long extinct and forgotten natural history society, the description of a little worm nicely corresponding to what we know today as *Caenorhabditis elegans*. The latter name was first introduced (as *Rhabditis elegans*) by Maupas in 1900 [14]. What should we do if the newly discovered paper containing an alternative name for the same worm, say *Rhabditis pusillus*, was dated, for example, 1876? Strict application of the Principle of Priority would require to stop using Maupas’ name, despite its immense popularity, to replace it with a completely forgotten senior synonym. To be sure, there would be plenty of reasons to make exception to the rule, in this particular case. However, problems with scientific names are too diverse to be automatically solved by the strict application of written rules, even if these are already detailed (and eventually cumbersome) as are the articles of the *Code* now in force. This is why specific, less tractable cases are regularly reported to the Commission for a ruling.

All these operations – those that can be definitely settled by strict application of the *Code* and also those that require intervention by the Commission – can be fixed only if the date at which the names enter zoological nomenclature can be determined unambiguously. But this is not that easy. To be available as the scientific name of an animal species, genus or family, a name must have been published (let’s ignore here the additional requirements fixed in the *Code* for different kinds of names). The name will take availability from the date at which the work was issued in which the name was proposed. The problem is, what ‘being published’ might actually mean today. This brings us straight to an amendment to the *Code* issued last September by the Commission [15]. These new rules are expected to profoundly affect the way the new scientific names of animals will be introduced from now on, with important although not strictly predictable consequences, positive but also potentially negative, on the stability of zoological nomenclature.

Accepting names introduced in online-only publications as valid for nomenclatural purposes is a welcome, but still cautious reply to what Charles Godfray [16] has described as the challenge for taxonomy in the present era dominated by information technology. The new amendment is not enough to ensure new prosperity, but to improve matters further I would suggest – as a mid-term strategy at least – a different target in respect to Godfray’s. His suggested strategy was centred on the use of the web to foster improvement and eventual fixation of taxonomy, together with the accompanying nomenclature. Realistically, Godfray imagined this to happen for different taxonomic groups independently. Thus, the taxonomy of, say, birds, or termites, or tapeworms, “could be self-contained and require reference to no other sources.” Unfortunately, this would work for taxonomy, but not for nomenclature. For example, the names of bird genera compete for priority with those of termites, tapeworms and all remaining animals, both extant and extinct, and problems of homonomy can be identified and eventually solved only in the context of the whole set of scientific names to which the *Code* applies. Therefore, despite the merits of Godfray’s plea for an increased use of the web as a forum where the exchange of data and interpretations can be easily made much more effective than it is today, progress with zoological nomenclature requires a different strategy. Information technology can be precious anyway, through an eventual improvement of the currently available tools, accompanied by a suitable change in the *Code*.

### Publishing a new animal name in the electronic era

The traditional concept of ’publication’, as fixed in the *Code*, was that of a work produced by a method assuring numerous identical and durable physical copies, i.e., printed on paper using ink or toner. This was arguably adequate until the ‘80s of the last century, but it is not so today, due to changed opportunities and practice in publication.

The need, or the opportunity, to consider also ‘electronic means’ of publication as valid for the purposes of zoological nomenclature was extensively debated some 15 years ago, when the Commission was preparing, in interaction with the zoological community worldwide, a new edition of the *Code*, eventually resulting in the version currently in force [2]. At the time, widespread concern was expressed about the qualities of the electronic means then available for the dissemination and storage of digital documents. This included concern about the inalterability of the documents themselves, about the future durability of their physical supports, and about the likely future availability of both hardware and software capable to retrieve the information stored in media and formats likely to disappear before long. A majority of commissioners, and of zoologists at large, agreed on a very cautious attitude towards accepting as valid publications also documents other than those issued on paper, although a window was kept open for future changes in the rules, whenever circumstances would allow more confidence in electronic media.

### Archives and registers of names

Discussion on electronically disseminated publications went largely in pace with discussion about the issue of *registration*. If scientific names are ruled by a code, why not to establish an official and publicly accessible repository of these names, and of associated information (e.g., full references to the publications where the names have been established)? About this issue, the zoological community (and the botanical alike) could look at two successful examples. One of these was provided by the international repositories of gene (or protein) sequences, such as GeneBank. The other was much closer to the needs of animal and plant taxonomists, as this international register was also one of names of species and higher taxa. This was the *Approved lists of bacterial names*[17,18]. Zoologists and botanists have always looked at this register, and the associated way of handling new names, with mixed feelings. On the one hand, the nomenclatural policy adopted by bacteriologists has easily proved successful in achieving those ideals of universality and uniqueness that also the zoological and botanical codes advocate as their most important inspiring principle. On the other, the huge number of plant and especially animal names, if compared to the much smaller number of names for bacterial taxa, suggested that to imitate bacteriologists would be much too difficult.

Practical difficulties notwithstanding, registration of names is an issue that cannot be dismissed too hastily.

Registration has two main aspects. One is to provide an archive of existing names, with all relevant associated information. At some date, this archive is closed: old names that have not been registered are as much as nonexistent, they will never threaten the stability of nomenclature anymore. The other side of registration is to provide a way to fix the availability of all the new names that will be introduced from now on. Under suitable, internationally agreed rules, registration may well be the best way to provide each name with an unambiguous date from which it takes availability – the basic requirement for any future application of the Principle of Priority.

But registration can be handled in different ways. We can register names and usages of names, or authors, or periodicals and books, or individual papers.

We can decide that the registration of a new name, or the registration of the publication where it is first introduced, is mandatory to confer availability to that name. Alternatively, we can simply recommend registration as this practice, even if facultative, is indeed a precious means to immediately inform all interested researchers about the existence of the new name.

There could be, however, a more radical use of a register of scientific names. Why shall we treat registration and effective publication of a new name as two distinct actions? Why not to use registration as the effective publication of the name, that is, as the action conferring availability to a new name?

### The new rules

Following year-long discussion, internal as well as external, on the Internet as well as in print, the Commission has recently decided to move ahead in the direction of registration as well as towards acknowledging the need to accept at least some forms of electronic publication as conferring availability to the new names and other nomenclatural acts contained therein.

A draft version of the amendment of the International Code of Zoological Nomenclature intended to expand and refine methods of publication was published already in 2008 [19]. However, the text eventually approved by the Commission [15], and now in force effective from the 1^st^ of January, 2012, is substantially different from the 2008 formulation, especially with regard to the requirement for registration of the publications containing nomenclatural acts, as explained below.

In the meantime, parallel, although not identical changes [20,21] have been also introduced for the scientific names of plants and fungi, with the adoption of a new edition of the botanical code, now titled *International Code of Nomenclature for algae, fungi, and plants (Melbourne Code)*, by the Eighteenth International Botanical Congress held in Melbourne, Australia, in July 2011 [22].

As for zoological nomenclature, the recent amendment to the *Code* has introduced the following important changes [15,23]. While the basic criteria remain, that a work must be (i) issued for the purpose of providing a public and permanent scientific record and (ii) obtainable, when first issued, free of charge or by purchase, and (iii) have been produced in an edition containing simultaneously obtainable copies – now these copies are not necessarily identified with physical copies (essentially, on paper). Following the amendment, the requirements for a *Code*-compliant publication are also satisfied by “widely accessible electronic copies with fixed content and layout”. However, works issued and distributed electronically are further subjected to the restrictive requirements of the new Art. 8.5, which is worth be reproduced here. To be considered published, a work issued and distributed electronically must

8.5.1. have been issued after 2011,

8.5.2. state the date of publication in the work itself, and

8.5.3. be registered in the Official Register of Zoological Nomenclature (ZooBank) (…) and contain evidence in the work itself that such registration has occurred. (…)

8.5.3.1. The entry in the Official Register of Zoological Nomenclature must give the name and Internet address of an organization other than the publisher that is intended to permanently archive the work in a manner that preserves the content and layout, and is capable of doing so. This information is not required to appear in the work itself.

8.5.3.2. The entry in the Official Register of Zoological Nomenclature must give an ISBN for the work or an ISSN for the journal containing the work. The number is not required to appear in the work itself.

8.5.3.3. An error in stating the evidence of registration does not make a work unavailable, provided that the work can be unambiguously associated with a record created in the Official Register of Zoological Nomenclature before the work was published.

Furthermore, with Art. 8.6 of the amendment a door remains open for future improvements of these rules, as *The Commission may issue Declarations to clarify whether new or unconventional methods of production, distribution, formatting or archiving can produce works that are published in the meaning of the Code.*

Besides the new technicalities with which taxonomists will be now confronted, these lines convey a very important and reassuring message: an Official Register of Zoological Nomenclature, or ZooBank [24], has been eventually established and entries are flowing into it. At the moment of writing, this archive includes entries for 35,931 publications, 17,055 authors and 88,383 nomenclatural acts. A massive effort will be required to register with ZooBank all relevant information for the few million names thus far proposed for animal taxa, and the corresponding authors and publications. But the system, at last, is working [25].

### Do the new rules offer a robust solution to the current needs of zoological nomenclature?

So far, so good. Troubles may derive, however, from a rule included in Art. 21.9 of the amendment: *Works issued on paper and electronically. A name or nomenclatural act published in a work issued in both print and electronic editions takes its date of publication from the edition that first fulfilled the criteria of publication of Article 8 and is not excluded by Article 9.*

Readers not familiar with the subtleties of the rules of zoological nomenclature are kindly invited to read these lines twice. To advocate priority to fix the publication date of a name first proposed in a paper issued in double edition (printed and online) is certainly in agreement with a fundamental principle of the *Code*. But let’s consider the issue more closely. Traditional papers issued only on paper have *one* date of publication. Today, as a rule, this date is explicitly (and perhaps honestly) specified in the paper itself. In some cases (much more frequently in older publications), the paper does not contain a precise indication of the day it was published, thus a bibliographic and/or archival search may be necessary to fix this date, to some approximation at least. A comparable degree of uncertainty could be totally extraneous to the newly issued publications, especially those that are issued online, with the date of publication literally embedded within them. Such precisely fixed dates of publication would be ideal for the application of the Principle of Priority. But this advantage in respect to former times, when the only publications existing (or the only accepted for the purposes of zoological nomenclature) were issued on paper, is potentially put at risk by a literal application of the new Art. 21.9, unless additional measures are taken either by the journal’s publisher, or by the author of the paper.

Some journals are published indeed both on paper and online, but their articles are issued in both editions exactly on the same day. This policy, for example, has been adopted by *Zootaxa* and *Zookeys* since these journals started publishing, thus long before the Commission issued the amendment on electronic publication. With printed and online editions published at the same time, there is no ambiguity in fixing the date of publication of a paper. Under these circumstances, nothing changes in effect with the new amendment, and a possible future restriction to online only will have no consequence for zoological nomenclature. It will be no surprise that *Zootaxa* is by far the most popular journal in zoological taxonomy (26,373 new taxa have been described in its pages between 2001 and 2012) and *Zookeys* is the journal that has offered till now the most important help in fostering registration of names (in addition to the now required registration of online-only papers) and thus a major supporter of ZooBank.

However, other journals may have, and actually have, a different policy for double publication. A small run of printed copies is usually sent out days or weeks after the publication of the paper online, and the printed copies are sometimes marked with a publication date other than the day the paper is released online. This opens full scope for the application of the new Art. 21.9. A rule is there, but its application is not necessarily straightforward. It requires a search, not necessarily an easy one, to ascertain whether a new name has been actually published twice rather than once and, if so, at which dates. As a consequence, in our times of transition from printed to digital documents, the new amendment may unwillingly open a new source of uncertainty and trouble.

In the first months, or years, of application of the amendment on electronic publications, we should also expect that a number of new names will be published online without complying with the additional requirements ensuring their availability in respect to the *Code*. For example, some publishers may issue papers containing the description of new species without registering those papers with ZooBank. The new names would thus be as much as non-existing for the purpose of zoological nomenclature, but the full descriptions accompanying them may tempt other unscrupulous ‘authors’ to publish them anew, with their own authorship, in hastily produced *Code*-compliant publications.

Worrying or disgusting as the practice to steal authorship of scientific names may be, this is not a new devious behaviour invited by the new way of producing and disseminating taxonomic information. Some forty years ago, when I was learning the first rudiments of taxonomic practice, my mentor Milo Burlini, a good specialist of chrysomelid beetles, told me a story. A dozen years earlier he had asked a senior specialist of the same insect group for advice about a little series of specimens he was inclined to describe himself as a new species. Several months of silence had followed: no reply to his enquiry. Eventually, however, he got from his correspondent a freshly published paper [26] where the old colleague described indeed Milo’s beetle as new, calling it *Pachybrachis fraudolentus*. Only the species’ sadly humorous epithet remains today to flag a story of stolen authorship. What can we do today to minimize or fully cancel the risk of similar misconduct?

## Conclusion

A few months ago, when the International Commission on Zoological Nomenclature voted on the amendment on electronic publications, I was one of the very few member to cast a negative vote. My main objection to the text eventually approved by a large majority was, simply, that the proposed change was not radical enough. Recognizing online publication as acceptable for the purpose of zoological nomenclature is certainly a positive momentous change, in respect to the fourth edition of the *Code*[2]. Momentous, but not strong enough.

Imagine a website providing access to all scientific names of animals proposed thus far and also offering an interactive interface through which you can enter all data (differential diagnosis or description, fixation of type etc.) required by the *Code* in order to make a new name available for the purposes of zoological nomenclature: all items, except for publication in the traditional sense of the word. Entering your data through that website would represent *registration as well as a publication*. The system should be careful instructed to check your input data for compliance with the *Code* and would offer you definitive assistance in avoiding the risk of proposing a junior homonym of an existing name. At the end of the registration/publication session, your new name would be not only available in the sense of the *Code*, but also immediately visible to everybody, thus strongly reducing the risk that other specialists working on the same animal group may long overlook the newly described species.

To be sure, implementing (and maintaining!) a similar website will not be that easy. Establishing such a system will require consummate skills in informatics to translate the articles of the *Code* into functions of the interactive website. It will also require a lot of time and money to fill the archive component of the system with a complete list of the scientific names of animals published to date. It will require steady financial support to ensure that it will function with the necessary continuity and safety.

In terms of resources involved, this would certainly be a ‘big science’ enterprise. In terms of its usefulness, not only for taxonomists, the producers of scientific names of organisms, but also for the users of names, this is a challenge to be seriously considered, technical and financial difficulties notwithstanding.

The ZooBank is a precious core out of which we may hope that a larger system such as outlined in the previous lines will eventually emerge. We must be very grateful to my fellow Commissioner Rich Pyle, of the Bishop Museum in Honolulu, for his unique commitment to the establishment of this repository of names and other information relevant to zoological nomenclature. Beyond its current precious functionalities, however, I regard ZooBank as the prototype of a comprehensive Website of Zoological Nomenclature through which we will eventually exploit the facilities of modern technology to improve the stability and universality of the scientific names of animals by implementing the conceptually simple identification of publication and registration of new names.

The Commission will hardly move further ahead in that direction without the help of the whole scientific community using zoological nomenclature. With the recent amendment, and with the establishment of ZooBank, the Commission has taken two important steps in the right direction. Let’s now help this old body steering the boat of nomenclature towards more ambitious but necessary targets, in pace with the spirit, the technology, and the needs of science in early twenty-first century.

## Competing interests

The views presented in this paper by a member (and former president) of the International Commission on Zoological Nomenclature express a personal perspective only, without any implied involvement of the Commission.
